# Fast 2D DOA Estimation Algorithm by an Array Manifold Matching Method with Parallel Linear Arrays

**DOI:** 10.3390/s16030274

**Published:** 2016-02-23

**Authors:** Lisheng Yang, Sheng Liu, Dong Li, Qingping Jiang, Hailin Cao

**Affiliations:** The State Key Laboratory of Aircraft Tracking Telemetering Command and Communication, Chongqing University, Chongqing 400044, China; yls@cqu.edu.cn (L.Y.); 20141213058@cqu.edu.cn (S.L.); jiangqp@cqu.edu.cn (Q.J.); hailincao@cqu.edu.cn (H.C.)

**Keywords:** 2D DOA estimation, array manifold matching, parallel linear arrays, automatic pairing

## Abstract

In this paper, the problem of two-dimensional (2D) direction-of-arrival (DOA) estimation with parallel linear arrays is addressed. Two array manifold matching (AMM) approaches, in this work, are developed for the incoherent and coherent signals, respectively. The proposed AMM methods estimate the azimuth angle only with the assumption that the elevation angles are known or estimated. The proposed methods are time efficient since they do not require eigenvalue decomposition (EVD) or peak searching. In addition, the complexity analysis shows the proposed AMM approaches have lower computational complexity than many current state-of-the-art algorithms. The estimated azimuth angles produced by the AMM approaches are automatically paired with the elevation angles. More importantly, for estimating the azimuth angles of coherent signals, the aperture loss issue is avoided since a decorrelation procedure is not required for the proposed AMM method. Numerical studies demonstrate the effectiveness of the proposed approaches.

## 1. Introduction

Direction-of-arrival (DOA) estimation plays an important role in many fields such as wireless communication, multiple-input multiple-output (MIMO) radar, sonar, *etc*. [1,2,3]. Many DOA estimation algorithms have been proposed to address the DOA estimation problem, for example, the multiple signal classification (MUSIC) algorithm [4,5], estimation of signal parameters via rotational invariance techniques (ESPRIT) algorithm [6] and propagator method (PM) [7]. Particularly the root-MUSIC algorithm, proposed in [5], can estimate more signals than elements. Based on these three classical algorithms, a large number of two-dimensional (2D) DOA estimation algorithms [8,9,10,11,12,13,14,15,16] were developed as well. Compared with the one-dimensional (1D) DOA estimation algorithms [4,5,6,7], the corresponding 2D DOA estimation algorithms face two difficulties, namely angle matching and increased complexity. Based on the assumption that the elevation and azimuth angles are independently estimated, many effective pair-matching methods were proposed [8,17,18]. For those methods, computational complexity is high, since twice 1D DOA estimation algorithms are involved. An algorithm called joint singular value decomposition (JSVD) [9] was proposed to achieve automatic pairing. However, this algorithm needs SVD of a high-order block covariance matrix, which is computationally demanding. The PM is a low-complexity DOA estimation algorithm because EVD or SVD is not required. In [15], an improved PM algorithm was proposed to achieve automatic pairing of the 2D DOA estimation. Compared with the original PM [10], this algorithm showed improved performance in both complexity and precision. But, in order to achieve automatic pairing, it needed to construct and compute a high-order correlation matrix, which increased computational complexity. In [19], a DOA matrix (DM) algorithm was presented in which elevation and azimuth angles were automatically paired. The DM, however, still performed twice eigenvalue decompositions (EVD).

It should be noted that the algorithms [4,5,6,7,8,9,10,11,12,13,14,15,16,19] were designed for incoherent signals. Unlike the incoherent signals, the rank of the covariance matrix of coherent signals is less than the number of incident signals. In order to overcome the rank deficiency, decorrelation approaches [20,21,22,23,24,25] were proposed. Apart from high complexity, these algorithms also suffered from aperture loss. In [20,21], a spatial smoothing (SS) technique was developed to decorrelate the incoming signals, but 2D peak searches are computationally intensive. In [22], an ESPRIT-like method was proposed for 2D DOA estimation of coherent signals with a rectangular array. For solving the problem of the rank deficiency, a pencil-based method was used to construct a high-order block Hankel matrix. However, the problem is still computationally demanding since it performed SVD of a high-order block matrix. In order to reduce the complexity, a unitary ESPRIT-like method [23] was presented based on the method [22]. By unitary transformation, EVD and SVD were transformed into real-valued process, but that only partially solved the problem. In [24], authors decorrelated the coherency of the signals and constructed the signal subspace using block covariance matrix (BCM). Compared with the traditional SS method, it showed improved performance in the case of low signal-to-noise ratio (SNR). However, the computational burden still is large due to the SVD of a high-order matrix as in [22]. In [25], fourth-order cumulants of received data from two-parallel uniform linear arrays were arranged to reconstruct two Toeplitz matrices (it is called the TMR algorithm in this paper), the rank of which is equal to the number of incoming signals. Although this algorithm had lower complexity than many similar algorithms, it caused more serious aperture loss than the SS and BCM methods.

In this paper, we propose two array manifold matching (AMM) methods using parallel linear arrays. The two methods are based on the assumption that the elevation angles are known or estimated. The first AMM method is designed for incoherent signals. By utilizing the estimated elevation angles, an array manifold matrix is constructed, which is used to eliminate the elevation angles in cross-covariance matrix, then to match and estimate the azimuth angles. This AMM method is called as unilateral AMM algorithm. The second AMM method is designed for coherent signals. Unlike the unilateral AMM algorithm, two array manifold matrices are constructed to match and estimate the azimuth angles. The second AMM method is referred to as bilateral AMM algorithm. The advantage of the proposed two AMM algorithms is that EVD or peak search is not needed. Moreover, the azimuth angles can be automatically matched with the estimated elevation angles. Combing the unilateral AMM method with PM [7], a low-complexity PM algorithm for incoherent signals is presented, which is called as PM-AMM. Computational complexity analysis shows that it is time efficient, but its estimation precision is still close to that of the PM [10,15]. Combing the bilateral AMM method with BCM method [24], a low-complexity ESPRIT-like for coherent signals is proposed, which is called as BCM-AMM. The elevation and azimuth angles are estimated by using SVD once in BCM-AMM. It demonstrates the lower complexity and the higher precision than the TMR algorithm [25]. The advantages of the proposed AMM algorithms are threefold. First, they can be used in conjunction with any 1D DOA estimation algorithms and the complexity is close to that 1D DOA estimation algorithm. Second, they can obtain automatically paired 2D DOA estimations. Finally, in the process of estimating azimuth angles for coherent signals, bilateral AMM algorithm does not cause aperture loss.

The rest of the paper is structured as follows: in Section 2, we introduce the signal model. In Section 3, we present the unilateral AMM algorithm for incoherent signals. In Section 4, we present the bilateral AMM algorithm for coherent signals. In Section 5, we present the application of AMM algorithm for L-shaped array. In Section 6, we present some simulation experiments to illustrate the validity of proposed algorithms. We give a summary of the paper in Section 7.

## 2. Signal Model and CRB

Parallel array [10,14,15,16,25] is one of commonly used planar arrays, and it plays an important role in 2D DOA estimation of multiple signals because of its simple geometry. Moreover, it also has many applications in other fields including multi-target localization by MIMO radar [2] and acoustics detection by vector sensor array [26]. In this section, we introduce the signal model of parallel array based on [10,14,15,16,25]. In addition, we suppose that mutual coupling is neglected for all proposed algorithms in this paper.

### 2.1. Signal Model of Parallel Array

**Figure 1:**
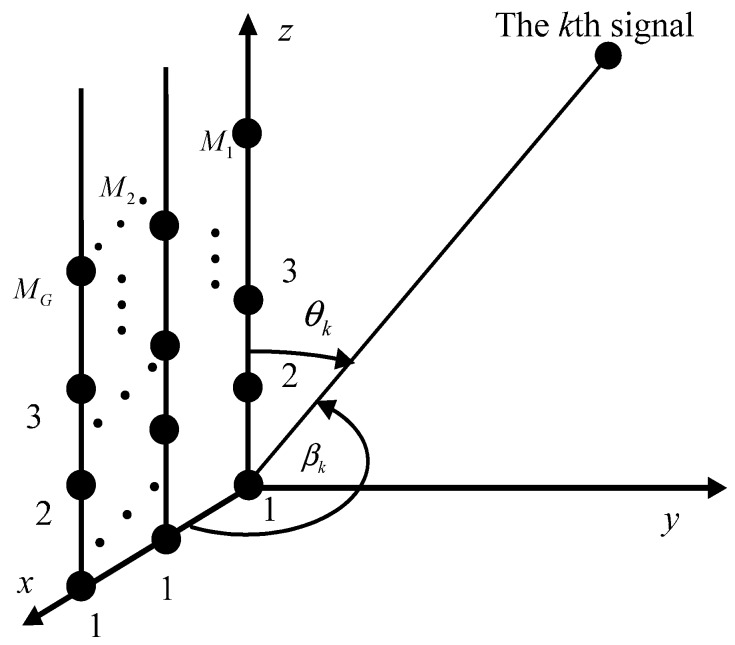
Array geometry of the *G* parallel linear arrays.

Consider an array consisting of *G* parallel uniform linear arrays. The array is located on *xoz* plane as shown in Figure 1, where the first linear array is located on the *z* axis. The coordinates of the sensors of the *g*th linear array successively are {(*g* − 1)*d*, 0, 0}, {(*g* − 1)*d*, 0, *d*},⋯,{(*g* − 1)*d*, 0, (*M_g_* − 1)*d*}, where *d* = *λ*/2 and *λ* is the wavelength of incident signals. Suppose that *K* far-field narrowband signals impinging on this array and let *θ_k_* and *β_k_* be the elevation angle and azimuth angle of the *k*th signal, respectively.

The observed vector at the *g*th linear array is $z_{g}(t)={\left[z_{g,1}(t),\cdots ,z_{g,M_{g}}(t)\right]}^{T}\in C^{M_{g}\times 1}$. With $\theta =\left[\theta_{1},\cdot \cdot \cdot ,\theta_{K}\right]$, $\beta =\left[\beta_{1},\cdot \cdot \cdot ,\beta_{K}\right]$, $z_{g}(t)$ now is

(1) $$z_{g}(t)=A_{g}(\theta )\Phi^{g-1}(\beta )s(t)+n_{g}(t)\;g=1,\cdots G;\;t=1,2\cdots T$$

where $s(t)={\left[s_{1}(t),\cdots ,s_{K}(t)\right]}^{T}\in C^{K\times 1}$ is the signal vector, $A_{g}(\theta )\Phi^{g-1}(\beta )\in C^{M_{g}\times K}$ is the manifold matrix of the *g*th linear array in which $A_{g}(\theta )=\left[a_{g}(\theta_{1}),\cdot \cdot \cdot ,a_{g}(\theta_{K})\right]\in C^{M_{g}\times K}$, $\Phi (\beta )=\text{diag}\left\{e^{-\frac{j2\pi d\;\text{cos}\;\beta_{1}}{\lambda }},e^{-\frac{j2\pi d\;\text{cos}\;\beta_{2}}{\lambda }},\cdot \cdot \cdot ,e^{-\frac{j2\pi d\;\text{cos}\;\beta_{K}}{\lambda }}\right\}\in C^{K\times K}$, and $a_{g}(\theta_{k})={\left[1,e^{-\frac{j2\pi d\;\text{cos}\;\theta_{k}}{\lambda }},\cdot \cdot \cdot ,e^{-\frac{j2\pi (M_{g}-1)d\;\text{cos}\;\theta_{k}}{\lambda }}\right]}^{T}\in C^{M_{g}\times 1}$ is the steering vector of the *g*th linear array to the *k*th signal, and $n_{g}(t)={\left[n_{g,1}(t),n_{g,2}(t),\cdots ,n_{g,M_{g}}(t)\right]}^{T}\in C^{M_{g}\times 1}$ is the noise vector in the *g*th linear array, which is assumed to be uncorrelated at different sensors.

### 2.2. CRB

The CRB is the performance benchmark for the estimation algorithms. The CRB of 2D DOA estimation with *G* parallel linear arrays is now derived. In [27], the CRB of 1D DOA estimation was analyzed. In [15], the CRB of 2D DOA estimation with two parallel linear arrays was developed. By utilizing the similar approach in [15,27], the CRB of 2D DOA estimation with *G* parallel linear arrays is obtained in this section. The received signal in Equation (1) can be reexpressed in a matrix form as:

(2) $$\left[\begin{array}{c} z_{1}(t)\\ \vdots \\ z_{G}(t) \end{array}\right]=\;W\;s(t)+\left[\begin{array}{c} n_{1}(t)\\ \vdots \\ n_{G}(t) \end{array}\right]$$

where $W={\left[A_{1}^{T}(\theta ),\cdots ,{\left(A_{G-1}(\theta )\Phi^{G-1}(\beta )\right)}^{T}\right]}^{T}\in C^{(M_{1}+\cdots +M_{G})\times K}$ aligns this equation.

With the signal model in Equation (2), the CRB is expressed as:

(3) $$\text{CRB}=\frac{\sigma^{2}}{2T}{\left\{\text{Re}\left[\left(D^{H}\Pi_{W}^{⊥}D\right)⊙{\hat{P}}^{T}\right]\right\}}^{-1}$$

where $\hat{P}=\left[\begin{array}{cc} {\hat{P}}_{S} & {\hat{P}}_{S}\\ {\hat{P}}_{S} & {\hat{P}}_{S} \end{array}\right]$, ${\hat{P}}_{S}=\frac{1}{T}\sum_{t=1}^{t=T}s(t)s^{H}(t)$, $w={\left[a_{1}^{T}(\theta ),\cdots ,e^{-\frac{j2\pi d(M_{G}-1)\;\text{cos}\;\beta }{\lambda }}a_{G}^{T}(\theta )\right]}^{T}\;\in C^{(M_{1}+\cdots +M_{G})\times 1}$, $\Pi_{W}^{⊥}=I_{M_{1}+\cdots +M_{G}}-W{\left(W^{H}W\right)}^{-1}W^{H}$, $D=\left[{\frac{\partial w}{\partial \theta }|}_{\theta =\theta_{1}},\cdots ,{\frac{\partial w}{\partial \theta }|}_{\theta =\theta_{K}},{\frac{\partial w}{\partial \beta }|}_{\beta =\beta_{1}},\cdots ,{\frac{\partial w}{\partial \beta }|}_{\beta =\beta_{K}}\right]$ and $\sigma^{2}$ is the power of noise.

In this paper, the two proposed AMM algorithms are based on the special parallel linear arrays with $M_{2}=M_{3}=\cdots =M_{G}$ and $A_{2}(\theta )=A_{3}(\theta )=\cdots =A_{G}(\theta )$. In order to facilitate representation, we let $M_{1}=M$, $M_{2}=M_{3}=\cdots =M_{G}=N$, $A_{1}(\theta )=A(\theta )$, $a_{1}(\theta )=a(\theta )$, $A_{2}(\theta )=A_{3}(\theta )=\cdots =A_{G}(\theta )=B(\theta )$ and $a_{2}(\theta )=a_{3}(\theta )=\cdots =a_{G}(\theta )=b(\theta )$ in the following sections. Particularly, when $M_{2}=M_{3}=\cdots =M_{G}=1$, the parallel linear arrays can be seen as an L-shaped array.

## 3. Unilateral AMM Algorithm for Incoherent Signals

In this section, we present the unilateral AMM algorithm for incoherent signals. For this AMM algorithm, the number of linear arrays should not be smaller than 3, namely G≥3. In Section 1, we mentioned that the AMM algorithm is based on the assumption that the elevation angles have been estimated. From Equation (1), we know that the vector $z_{1}(t)$ only contains the information of elevation angles. Therefore, existing 1D DOA estimation algorithms can be adopted to estimate the elevation angles with vector $z_{1}(t)$. We now use the low-complexity PM [7] as an example to verify the availability of the unilateral AMM algorithm.

### 3.1. The Estimation of Elevation by PM Algorithm

The correlation matrix of the first linear array and other arrays is defined by $C_{g}=E\left\{z_{1}z_{g}^{H}\right\}\in C^{M\times N}$, g=2,3,⋯,G. Since the noises of different sensors are uncorrelated, the correlation matrix is given by:

(4) $$C_{g}=E\left\{z_{1}z_{g}^{H}\right\}=AR_{s}{\left[\Phi^{g-1}(\beta )\right]}^{H}B^{H}$$

Specially, the correlation matrix of the first linear array and the second linear array is:

(5) $$C_{2}=E\left\{z_{1}z_{2}^{H}\right\}=AR_{s}\Phi^{H}(\beta )B^{H}$$

where $R_{s}=E\left\{s(t)s^{H}(t)\right\}=diag\left\{p_{1},p_{2},\cdots ,p_{K}\right\}$.

Partitioning the matrix **A** into two part yields:

(6) $$A=\left[\begin{array}{c} A_{1}\\ A_{2} \end{array}\right]$$

where $A_{1}\in C^{K\times K}$ is the submatrix containing the first *K* rows of **A** and $A_{2}\in C^{(M-K)\times K}$ is the submatrix containing the remaining *M*-*K* rows of **A**. It is easy to determine that $A_{1}$ is a nonsingular matrix, which means there must be a matrix $P\in C^{(M-K)\times K}$ such that:

(7) $$PA_{1}=A_{2}$$

Similarly, partitioning the matrix **C**_2_ gives:

(8) $$C_{2}=\left[\begin{array}{c} C_{21}\\ C_{22} \end{array}\right]=\left[\begin{array}{c} A_{1}R_{s}\Phi^{H}(\beta )B^{H}\\ A_{2}R_{s}\Phi^{H}(\beta )B^{H} \end{array}\right]=\left[\begin{array}{c} A_{1}R_{s}\Phi^{H}(\beta )B^{H}\\ PA_{1}R_{s}\Phi^{H}(\beta )B^{H} \end{array}\right]$$

where $C_{21}\in C^{K\times N}$ contains the first *K* rows of $C_{2}$ and $C_{22}\in C^{(M-K)\times N}$ contains the remaining *M*-*K* rows of $C_{2}$. It is established that $C_{21}$ is a row full-rank matrix [7].

Utilizing Equation (7), the relationship of **C**_21_ and **C**_22_ is:

(9) $$PC_{21}=C_{22}$$

Since **C**_21_ is a row full-rank matrix, **P** is obtained as $P=C_{22}{\left(C_{21}\right)}^{+}$. Denoting $P_{0}=\left[\begin{array}{c} I_{K}\\ P \end{array}\right]$, we can obtain $P_{0}A_{1}=A$. Let $P_{1}$ contain the first *M*-1 rows of $P_{0}$, and $P_{2}$ contain the last *M*-1 rows of $P_{0}$. Utilizing PM algorithm, we have:

(10) $$A_{1}\Omega (\theta ){\left(A_{1}\right)}^{-1}={\left(P_{1}\right)}^{+}P_{2}$$

where $\Omega (\theta )=\text{diag}\left\{e^{-\frac{j2\pi d\;\text{cos}\;\theta_{1}}{\lambda }},e^{-\frac{j2\pi d\;\text{cos}\;\theta_{2}}{\lambda }},\cdot \cdot \cdot ,e^{-\frac{j2\pi d\;\text{cos}\;\theta_{K}}{\lambda }}\right\}$.

The estimation of **θ** is now can be obtained by performing the EVD of ${\left(P_{1}\right)}^{+}P_{2}$ [15].

**Remark** **1.**  From Equations (4)–(10), it is noted that this PM algorithm is based on the cross-covariance matrix of the received vectors from two different subarrays, which is different from Wu’s PM [10] and Li’s PM [15]. In order to achieve angle matching, Wu’s PM and Li’s PM used the covariance matrix of the received vector from whole array. That means the order of the covariance matrix is much higher than the cross-covariance matrix **C**_2_.

### 3.2. Unilateral AMM Algorithm for the Estimation of Azimuth Angle

According to Equations (4) and (5), a partitioned matrix $C\in C^{M\times (G-1)N}$ is defined as:

(11) $$C=\left[\begin{array}{cccc} C_{2} & C_{3} & \cdots & C_{G} \end{array}\right]=\left[\begin{array}{cccc} AR_{s}\Phi^{H}B^{H} & AR_{s}{\left(\Phi^{2}\right)}^{H}B^{H} & \cdots & AR_{s}{\left(\Phi^{G-1}\right)}^{H}B^{H} \end{array}\right]=AR_{s}\left[\begin{array}{cccc} \Phi^{H}B^{H} & {\left(\Phi^{2}\right)}^{H}B^{H} & \cdots & {\left(\Phi^{G-1}\right)}^{H}B^{H} \end{array}\right]$$

Suppose that $\hat{\theta }=[{\hat{\theta }}_{e1},{\hat{\theta }}_{e2},\cdots ,{\hat{\theta }}_{eK}]$ is the estimation of **θ**, where the arrangements of ${\hat{\theta }}_{e1},{\hat{\theta }}_{e2},\cdots ,{\hat{\theta }}_{eK}$ are arbitrary. Let $\hat{A}$ be the manifold matrix, denoted by $\hat{A}=[a({\hat{\theta }}_{e1}),a({\hat{\theta }}_{e2}),\cdots ,a({\hat{\theta }}_{eK})]$, and it is easy to show that $\hat{A}$ is a column full-rank matrix. Then, we have:

(12) $${\left[{\hat{A}}^{+}\right]}_{k,:}{\left[\hat{A}\right]}_{:,j}=\{\begin{array}{c} 0;k\neq j\\ 1;k=j \end{array}$$

Assume that ${\hat{\theta }}_{ek}$ is the estimation of $\theta_{t}$, and we can derive:

(13) $${\left[{\hat{A}}^{+}\right]}_{k,:}{\left[A\right]}_{:,j}={\left[{\hat{A}}^{+}\right]}_{k,:}a(\theta_{j})\approx \{\begin{array}{c} 0;j\neq t\\ 1;j=t \end{array}\;j=1,2,\cdots ,K$$

According to Equation (13), we have:

(14) $${\left[{\hat{A}}^{+}\right]}_{k,:}C={\left[{\hat{A}}^{+}\right]}_{k,:}A\text{diag}\left\{p_{1},p_{2},\cdots ,p_{K}\right\}\left[\begin{array}{cccc} \Phi^{H}B^{H} & {\left(\Phi^{2}\right)}^{H}B^{H} & \cdots & {\left(\Phi^{G-1}\right)}^{H}B^{H} \end{array}\right]={\left[{\hat{A}}^{+}\right]}_{k,:}\left[a(\theta_{1}),\cdot \cdot \cdot ,a(\theta_{K})\right]\text{diag}\left\{p_{1},p_{2},\cdots ,p_{K}\right\}\left[\begin{array}{cccc} \Phi^{H}B^{H} & {\left(\Phi^{2}\right)}^{H}B^{H} & \cdots & {\left(\Phi^{G-1}\right)}^{H}B^{H} \end{array}\right]\approx \left[0,\cdots ,\underset{\text{the}\;t\text{th}\;\text{element}}{\underset{︸}{1}},\cdots 0\right]\text{diag}\left\{p_{1},p_{2},\cdots ,p_{K}\right\}\left[\begin{array}{cccc} \Phi^{H}B^{H} & {\left(\Phi^{2}\right)}^{H}B^{H} & \cdots & {\left(\Phi^{G-1}\right)}^{H}B^{H} \end{array}\right]=\left[0,\cdots ,\underset{\text{the}\;t\text{th}\;\text{element}}{\underset{︸}{p_{t}}},\cdots 0\right][\begin{array}{cccc} \Phi^{H}B^{H} & {\left(\Phi^{2}\right)}^{H}B^{H} & \cdots & {\left(\Phi^{G-1}\right)}^{H}B^{H} \end{array}]=p_{t}{\left[\begin{array}{cccc} \Phi^{H}B^{H} & {\left(\Phi^{2}\right)}^{H}B^{H} & \cdots & {\left(\Phi^{G-1}\right)}^{H}B^{H} \end{array}\right]}_{t,:}=p_{t}\left[\begin{array}{cccc} e^{\frac{j2\pi d\;\text{cos}\;\beta_{t}}{\lambda }}b^{H}(\theta_{t}) & e^{\frac{j4\pi d\;\text{cos}\;\beta_{t}}{\lambda }}b^{H}(\theta_{t}) & \cdots & e^{\frac{j2\pi (G-1)d\;\text{cos}\;\beta_{t}}{\lambda }}b^{H}(\theta_{t}) \end{array}\right]$$

Since *p_t_* is constant, from Equation (14), we have:

(15) $$e^{-\frac{j2\pi d\;\text{cos}\;\beta_{t}}{\lambda }}=\frac{{\left[\begin{array}{cccc} e^{\frac{j2\pi d\;\text{cos}\;\beta_{t}}{\lambda }}b^{H}(\theta_{t}) & e^{\frac{j4\pi d\;\text{cos}\;\beta_{t}}{\lambda }}b^{H}(\theta_{t}) & \cdots & e^{\frac{j2\pi (G-1)d\;\text{cos}\;\beta_{t}}{\lambda }}b^{H}(\theta_{t}) \end{array}\right]}_{n}}{{\left[\begin{array}{cccc} e^{\frac{j2\pi d\;\text{cos}\;\beta_{t}}{\lambda }}b^{H}(\theta_{t}) & e^{\frac{j4\pi d\;\text{cos}\;\beta_{t}}{\lambda }}b^{H}(\theta_{t}) & \cdots & e^{\frac{j2\pi (G-1)d\;\text{cos}\;\beta_{t}}{\lambda }}b^{H}(\theta_{t}) \end{array}\right]}_{N+n}}\approx \frac{{\left({\left[{\hat{A}}^{+}\right]}_{k,:}C\right)}_{n}}{{\left({\left[{\hat{A}}^{+}\right]}_{k,:}C\right)}_{N+n}},\;n=1,2,\cdots ,(G-2)N$$

The estimation of $\beta_{t}$ now can be obtained using Equation (15) as:

(16) $${\hat{\beta }}_{t}=\text{arccos}\left\{-\frac{\lambda }{2\pi d}\text{angle}\left[\frac{1}{(G-2)N}\sum_{n=1}^{(G-2)N}\frac{{\left({\left[{\hat{A}}^{+}\right]}_{k,:}C\right)}_{n}}{{\left({\left[{\hat{A}}^{+}\right]}_{k,:}C\right)}_{N+n}}\right]\right\}$$

We know ${\hat{\beta }}_{t}$ is matched with ${\hat{\theta }}_{ek}$ because it is obtained based on the assumption that ${\hat{\theta }}_{ek}$ is the estimation of $\theta_{t}$. According to Equations (11)–(16), it is seen that the estimator Equation (16) is related to the estimated elevation angles $\hat{\theta }$, but it does not require the method of obtaining the elevation angles $\hat{\theta }$. This is the reason why the elevation angles can be estimated by any 1D DOA estimation algorithms. Hence, the proposed unilateral AMM algorithm can be combined with arbitrary 1D estimator such as [4,5,6,7]. Because of the similarity in the principle, we only take the PM as an example to avoid redundancy. In this section, we only consider the case of *G* = 3.

### 3.3. The Selection of M, N

From Section 3.1, we know that the estimation accuracy of elevation angles is affected by the value of *M*. From Section 3.2, we also know that the estimation accuracy of azimuth angles is affected by the value of *N*. In addition, it should be noticed that the azimuth angles are obtained by estimated elevation angles. Hence, the accuracy of elevation angles also affects the accuracy of azimuth angles. It is difficult to determine the exact relationship between *M* and *N*, but after intensive experiments, the three-parallel linear array with *M* > *N* is chosen. To produce satisfactory performance, *N* should not be too small. In Section 6, the results of the first simulation experiment can roughly demonstrate the validity of this selection. Although we are unable to obtain the exact values of *M* and *N*, an approximate range is that *N* should be close to *M*/2.

### 3.4. Complexity Analysis

**Figure 2:**
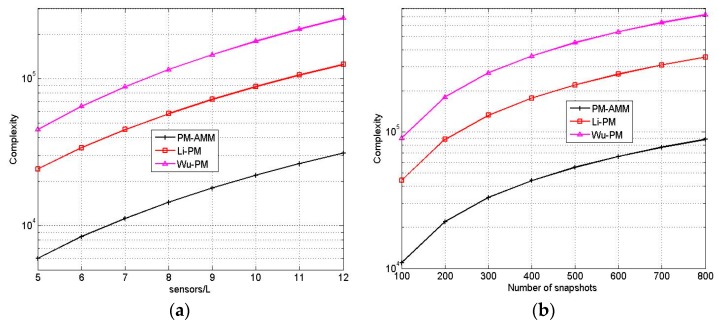
Complexity comparison of three algorithms. (**a**) T = 200; (**b**) L = 10.

In Section 1, we have introduced many PM algorithms [10,11,12,13,14,15], where the algorithms [11,12,13,14] are based on L-shaped array and the algorithms [10,15] are based on two parallel arrays. Hence, we only compare the proposed PM-AMM to Wu’s PM [10] and Li’s PM [15] in this subsection. With T≫M,K, and the complexity of proposed PM-AMM is *O*{2*NMT*}. Suppose the number of elements for Li’s PM [15] and Wu’s PM [10] is 2*L* + 1, the complexity of Li’s PM is *O*{[2*L*+1]^2^*T*} and the complexity of Wu’s PM is *O*{(3*L*)^2^*T*}. To guarantee the same number of elements, if *L* is odd number, we let *M* = *L* +1 and *N* = *L*/2, and if *L* is even number, we let *M* = *L* and *N* = (*L* + 1)/2. Therefore, the complexity of proposed PM-AMM is *O*{(*L*+1)*LT*}. The complexity comparison *versus* different *L* and *T* is provided in Figure 2. It is observed that the complexity of proposed PM-AMM is far lower than that of Li’s PM and Wu’s PM. It is in the agreement with the theoretical analysis.

## 4. Bilateral AMM Algorithm for Correlated Signals

In this section, we develop the bilateral AMM algorithm for correlated signals using parallel linear arrays. For this AMM algorithm, the number of linear arrays should not be smaller than 2, namely G≥2. The principle of bilateral AMM algorithm is similar to the unilateral AMM algorithm proposed in Section 3. We also need to use an existing method to estimate the elevation angles of the correlated signals. Here, we adopt the BCM-based ESPRIT-like [24] to estimate the elevation angles. Then we develop the bilateral AMM algorithm to estimate the azimuth angles of correlated signals.

### 4.1. The Estimation of Elevation by BCM-Based ESPRIT-Like Algorithm

In this case, the correlation matrix is denoted by $C_{g}=E\left\{z_{1}z_{g}^{H}\right\}\in C^{M\times N}$, g=2,3,⋯,G as in Section 3. Similarly, we have:

(17) $$C_{g}=E\left\{z_{1}z_{g}^{H}\right\}=AR_{s}{\left[\Phi^{g-1}(\beta )\right]}^{H}B^{H}$$

We assume that the number of signals *K* and the number of coherent group *q* are known, also we assume signals in the same group are coherent, but uncorrelated with the signals in other groups. Without loss of generality, assume the largest coherent group contains *L_max_* coherent signals, and then we use the **C**_2_ to reconstruct a partitioned matrix ${\bar{C}}_{2}\in C^{(M+1-L_{\text{max}})\times NL_{\text{max}}}$ as [24]:

(18) $${\bar{C}}_{2}=\left[C_{21},C_{22},\cdots ,C_{2L_{\text{max}}}\right]$$

where $C_{2l}\in C^{(M+1-L_{\text{max}})\times N}$, $l=1,2,\cdots ,L_{\text{max}}$ is:

(19) $$C_{2l}={\left[C_{2}\right]}_{l:M-L_{\text{max}}+l,:}$$

It is easy to determine $\text{rank}({\bar{C}}_{2})=K$. Using the ESPRIT-like algorithm in [24], estimations of elevation angles are produced by SVD of the ${\bar{C}}_{2}$.

We should point out that the BCM method is similar to forward/backward spatial smoothing (SS) technique. However, compared with the SS method, it showed improved performance in the case of low SNR.

### 4.2. Bilateral AMM Method for the Estimation of Azimuth Angle

From Equation (17), the diagonal elements of matrix **R***_s_* are the powers of the *K* signals. In the general case, the **R***_s_* is expressed as:

(20) $$R_{S}=\left[\begin{array}{cccc} p_{11} & p_{12} & \cdots & p_{1K}\\ p_{21} & p_{22} & \cdots & p_{2K}\\ \vdots & \vdots & \ddots & \vdots \\ p_{K1} & p_{K2} & \cdots & p_{KK} \end{array}\right]$$

where $p_{kk},\;k=1,2,\cdots K$ denotes the power of the *k*th signal, and it is a real number.

With Equation (20), $C_{g}$ now is expressed as:

(21) $$C_{g}=A\left[\begin{array}{ccc} p_{11}e^{\frac{j2\pi (g-1)d\;\text{cos}\;\beta_{1}}{\lambda }} & \cdots & *\\ \vdots & \ddots & \vdots \\ {}* & \cdots & p_{KK}e^{\frac{j2\pi (g-1)d\;\text{cos}\;\beta_{K}}{\lambda }} \end{array}\right]B^{H},\;g=2,3,\cdots ,G$$

where “*” stands for the unknown element.

Similarly as in Section 3.2, with the notations of $\hat{\theta }=\left[{\hat{\theta }}_{e1},{\hat{\theta }}_{e2},\cdots ,{\hat{\theta }}_{eK}\right]$, $\hat{A}=\left[a({\hat{\theta }}_{e1}),a({\hat{\theta }}_{e2}),\cdots ,a({\hat{\theta }}_{eK})\right]$, $\hat{B}=\left[b({\hat{\theta }}_{e1}),b({\hat{\theta }}_{e2}),\cdots ,b({\hat{\theta }}_{eK})\right]$ and assume that ${\hat{\theta }}_{e_{k}}$ is the estimation of $\theta_{t}$, and we have:

(22) $${\left[{\hat{A}}^{+}\right]}_{k,:}{\left[A\right]}_{:,i}\{\begin{array}{c} \approx 1,\;i=t\\ \approx 0,\;i\neq t \end{array}$$

(23) $${\left[{\hat{B}}^{+}\right]}_{k,:}{\left[B\right]}_{:,i}\{\begin{array}{c} \approx 1,\;i=t\\ \approx 0,\;i\neq t \end{array}$$

From Equations (22) and (23), for any g=2,3,⋯,G, we have:

(24) $${\left[{\hat{A}}^{+}\right]}_{k,:}C_{g}{\left({\left[{\hat{B}}^{+}\right]}_{k,:}\right)}^{H}={\left[{\hat{A}}^{+}\right]}_{k,:}A\left[\begin{array}{ccc} p_{11}e^{\frac{j2\pi (g-1)d\;\text{cos}\;\beta_{1}}{\lambda }} & \cdots & *\\ \vdots & \ddots & \vdots \\ {}* & \cdots & p_{KK}e^{\frac{j2\pi (g-1)d\;\text{cos}\;\beta_{K}}{\lambda }} \end{array}\right]B^{H}{\left({\left[{\hat{B}}^{+}\right]}_{k,:}\right)}^{H}\approx \left[0,\cdots ,\underset{\text{the}\;t\text{th}\;\text{element}}{\underset{︸}{1}},\cdots 0\right]\left[\begin{array}{ccc} p_{11}e^{\frac{j2\pi (g-1)d\;\text{cos}\;\beta_{1}}{\lambda }} & \cdots & *\\ \vdots & \ddots & \vdots \\ {}* & \cdots & p_{KK}e^{\frac{j2\pi (g-1)d\;\text{cos}\;\beta_{K}}{\lambda }} \end{array}\right]{\left[0,\cdots ,\underset{\text{the}\;t\text{th}\;\text{element}}{\underset{︸}{1}},\cdots 0\right]}^{H}=p_{tt}e^{\frac{j2\pi (g-1)d\;\text{cos}\;\beta_{t}}{\lambda }}$$

Utilizing $w_{1}=1$ and $w_{g}={\left[{\hat{A}}^{+}\right]}_{k,:}C_{g}{\left({\left[{\hat{B}}^{+}\right]}_{k,:}\right)}^{H}$, g=2,3,⋯,G, and based on Equation (24), we have:

(25) $$w_{g}/w_{g-1}\{\begin{array}{ccc} =e^{\frac{j2\pi d\;\text{cos}\;\beta_{t}}{\lambda }}, & & g=3,\cdots ,G\\ =p_{tt}e^{\frac{j2\pi d\;\text{cos}\;\beta_{t}}{\lambda }}, & & g=2 \end{array}$$

Since *p_tt_* is a real number, utilizing Equation (25) produces estimation of $\beta_{t}$, given by:

(26) $${\hat{\beta }}_{t}=\text{arccos}\left\{\frac{\lambda }{2\pi d}\frac{1}{G-1}\left[\sum_{g=2}^{G}\text{angle}\left(\frac{w_{g}}{w_{g-1}}\right)\right]\right\}$$

The estimate of ${\hat{\beta }}_{t}$ is matched with ${\hat{\theta }}_{ek}$ since it is obtained based on the assumption that ${\hat{\theta }}_{ek}$ is the estimation of $\theta_{t}$. From Equations (20)–(26), it is seen that the estimator Equation (26) also is related to the estimated elevation angles $\hat{\theta }$, but it does not need to know how to obtain the elevation angles $\hat{\theta }$. Hence, this AMM algorithm also can be applied to any 1D DOA estimation algorithms of correlated signals. In this section, we only consider *G* = 2, 3 in the following sections.

**Remark** **2.**  For many DOA estimation algorithms of coherent signals using decorrelation approach, loss of aperture is a highlighted weakness. However, the estimator Equation (26) will not cause aperture loss. From Equations (20)–(26), we also find the bilateral AMM method is suitable for incoherent signals.

### 4.3. Complexity Analysis

In Section 1, we have discussed several DOA algorithms [20,21,22,23,24,25] for coherent signals, where the algorithms [20,21,22,23] are based on rectangular array, the algorithm [24] is based on two L-shaped arrays and the algorithm [25] is based on two parallel arrays. Hence, in this work, we compare the complexity of proposed BCM-AMM algorithm with TMR [25]. For a fair comparison, both algorithms use two (2*L* + 1)-element parallel linear arrays, where T≫2L+1. The main complexity of TMR [25] is $O\{18{\left(L+1\right)}^{2}T+2{\left(L+1\right)}^{3}\}$. The main complexity of the proposed BCM-AMM algorithm is $O\{{\left(2L+1\right)}^{2}T+{\left(2L+1-l_{\text{max}}\right)}^{3}\}$.

**Figure 3:**
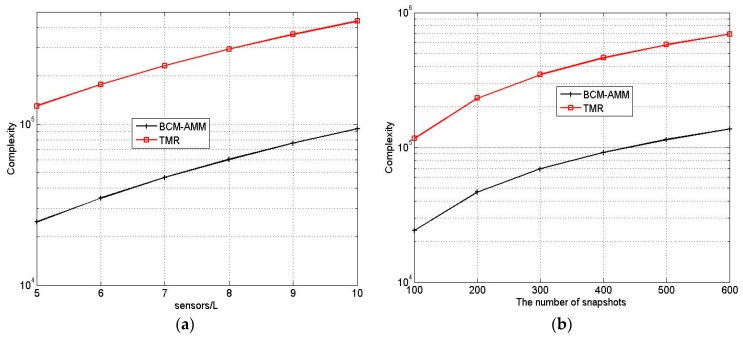
Complexity comparison of two algorithms. (**a**) T = 200; (**b**) L = 7.

The complexity comparison *versus* different *L* and *T* with $l_{\text{max}}=3$ is provided in Figure 3. It shows that the complexity of proposed BCM-AMM algorithm is far lower than that of the TMR, which agrees with our theoretical analysis.

## 5. AMM Algorithm for L-Shaped Array

From Section 2, we can know that the parallel linear array can be seen as an L-shaped array with $M_{2}=M_{3}=\cdots =M_{G}=1$. In order to make the proposed AMM algorithm more convincing, we combine the AMM with JSVD and ESPRIT algorithm and analyse the improved performance in complexity.

We use JSVD [9] algorithm to estimate elevation angles and use proposed AMM algorithm to estimate azimuth angles (we call this algorithm as JSVD-AMM). We use ESPRIT [6] algorithm to estimate elevation angles and use proposed AMM algorithm to estimate azimuth angles (we call this algorithm as ESPRIT-AMM). Consider an L-shaped array consisting of two linear arrays, namely, $M_{1}=L$ and $M_{2}=M_{3}=\cdots =M_{L+1}=1$. This array configuration is the same as the array used in JSVD [9] and CCM-ESPRIT [17]. The main complexity of JSVD is $O\{L^{2}T+{\left(2L\right)}^{3}+2LK\eta \}$, where η is the number of scanning. The main complexity of the JSVD-AMM algorithm is $O\{L^{2}T+{\left(2L\right)}^{3}\}$. The main complexity of CCM-ESPRIT is $O\{2L^{2}T+2L^{3}+2LKT\}$. The main complexity of the ESPRIT-AMM algorithm is $O\{L^{2}T+L^{3}\}$. Obviously, JSVD-AMM is more effective than JSVD and ESPRIT-AMM is more effective than CCM-ESPRIT.

## 6. Simulation Results

**Figure 4:**
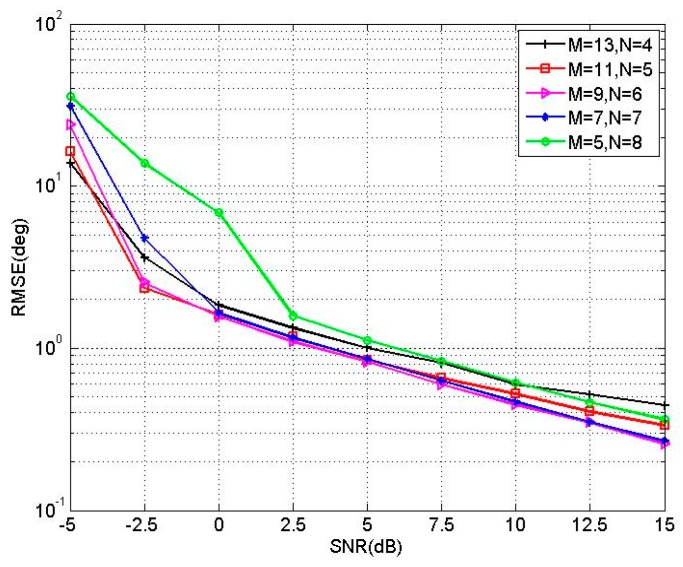
RMSE of different *M* and *N versus* SNR.

In this section, totally seven experiments are presented to demonstrate the effectiveness of proposed algorithms. The root-mean-square error (RMSE) of DOA estimation as the performance measure is given by:

(27) $$\text{RMSE}=\sqrt{\frac{1}{JK}\sum_{k=1}^{K}\sum_{j=1}^{J}{\left({\hat{\theta }}_{jk}-\theta_{k}\right)}^{2}+{\left({\hat{\beta }}_{jk}-\beta_{k}\right)}^{2}}$$

where J=500, and ${\hat{\theta }}_{jk}$, ${\hat{\beta }}_{jk}$ are the estimations of the *k*th signal in the *j*th Monte Carlo trial. In the first experiment, we compare the performance of the proposed PM-AMM with three-parallel array for different values of *M*, *N*. Two uncorrelated sources are located at the angles of $\left[\theta_{1},\theta_{2}]=[50^{\circ },60^{\circ }\right]$, $\left[\beta_{1},\beta_{2}]=[20^{\circ },30^{\circ }\right]$ and suppose *M* + 2*N* = 21. The RMSEs of different *M*, *N versus* SNR with T = 200 are provided in Figure 4.

It is noted that the estimation performance when *M* = 11, *M* = 9 and *M* = 7 is better than that when *M* = 13, *M* = 5. In addition, the performance of *M* = 11 is slightly better than that of *M* = 7 and *M* = 9 when SNR is low. The results approximately support our viewpoint in Section 3.3 on the value selection of *M* and *N*. In the following two experiments, the parameter set of *M* = 11, *N* = 5 is chosen.

**Figure 5:**
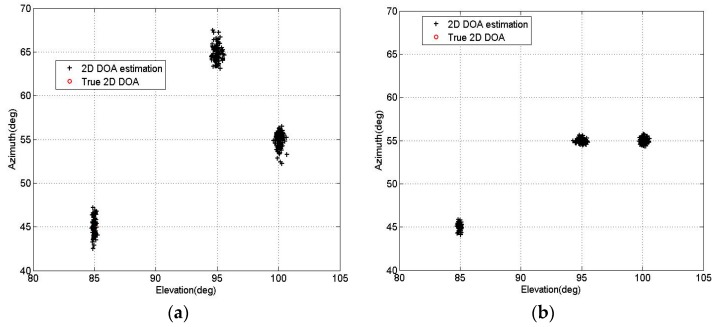
2D DOA estimation results of PM-AMM algorithm. (**a**) Three signals with different elevation and azimuth angles; (**b**) Two signals with the same azimuth angles.

In the second experiment, the pairing effectiveness and resolution of the PM-AMM algorithm are demonstrated. We use a three-parallel array with *M* = 11, *N* = 5, and consider three uncorrelated sources located at the angles of $\left[\theta_{1},\theta_{2},\theta_{3}]=[85^{\circ },95^{\circ },100^{\circ }\right]$, $\left[\beta_{1},\beta_{2},\beta_{3}]=[45^{\circ },65^{\circ },55^{\circ }\right]$. Figure 5a depicts the 2D DOA estimation results of the proposed PM-AMM with T = 200 and SNR = 10 dB. Now we keep the same elevation angles and change the azimuth angles to $\left[\beta_{1},\beta_{2},\beta_{3}]=[45{}^{\circ},55{}^{\circ},55{}^{\circ}\right]$. Figure 5b depicts the 2D DOA estimation results of the PM-AMM with T = 200 and SNR = 10 dB under the new azimuth angles. From both the figures, the elevation and azimuth angles can be clearly observed and correctly matched. Particularly, the proposed PM-AMM algorithm is able to separate the signals with the same azimuth angles.

**Figure 6:**
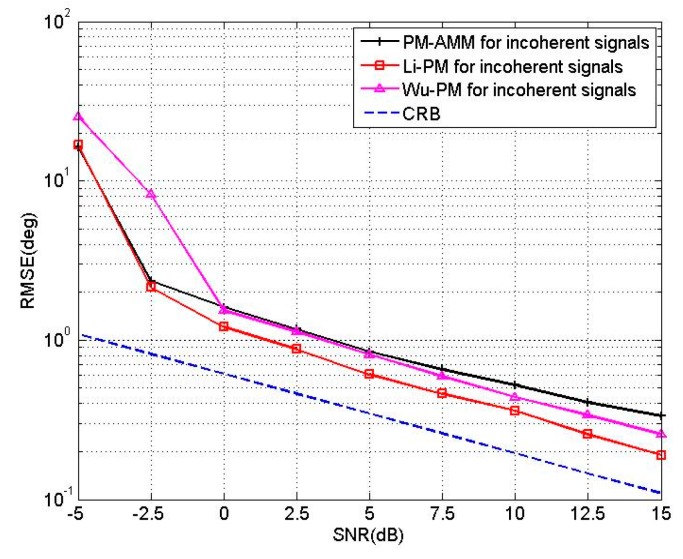
RMSE of PM-AMM, Li’PM and Wu’PM *versus* SNR.

**Figure 7:**
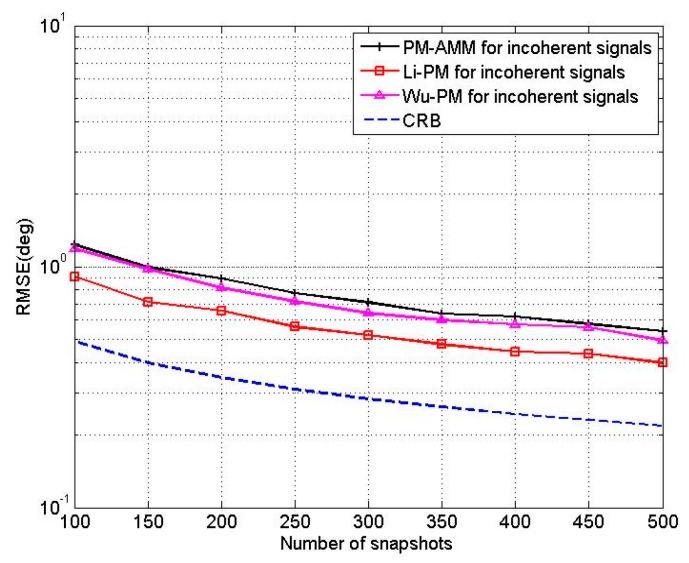
RMSE of PM-AMM, Li’s PM and Wu’s PM *versus* snapshots.

In the third experiment, we compare the proposed PM-AMM algorithm with Wu’s PM [10], Li’s PM [15] and CRB. A three-parallel array with *M* = 11, *N* = 5 is used, and two uncorrelated sources are located at the angles of $\left[\theta_{1},\theta_{2}]=[50{}^{\circ},60{}^{\circ}\right]$, $\left[\beta_{1},\beta_{2}]=[20{}^{\circ},30{}^{\circ}\right]$. For the Wu’s PM [10] and Li’s PM [15], we use an 11-element linear array and a 10-element linear array, respectively. Figure 6 shows the RMSEs of the proposed PM-AMM, Wu’s PM and Li’s PM *versus* SNR with T = 200. Figure 7 shows the RMSEs of proposed PM-AMM, Wu’s PM and Li’s PM *versus* snapshots with SNR = 5 dB. Inspecting both figures shows that the estimation precision of the proposed PM-AMM is close to that of Li’s PM and Wu’s PM. Keep in mind that from the complexity analysis, the complexity of the proposed PM-AMM is far lower than that of Li’s PM and Wu’s PM. Therefore, the PM-AMM is an attractive option to practical uses.

In the fourth experiment, the pairing effectiveness and resolution of the BCM-AMM algorithm are demonstrated for coherent signals. A two-parallel array with *M* = 15, *N* = 15 is used, and three sources are located at the angles of $\left[\theta_{1},\theta_{2},\theta_{3}]=[80{}^{\circ},85{}^{\circ},90{}^{\circ}\right]$, $\left[\beta_{1},\beta_{2},\beta_{3}]=[47.5{}^{\circ},45{}^{\circ},50{}^{\circ}\right]$, where the first and third signals are coherent and they are uncorrelated with the second signal.

**Figure 8:**
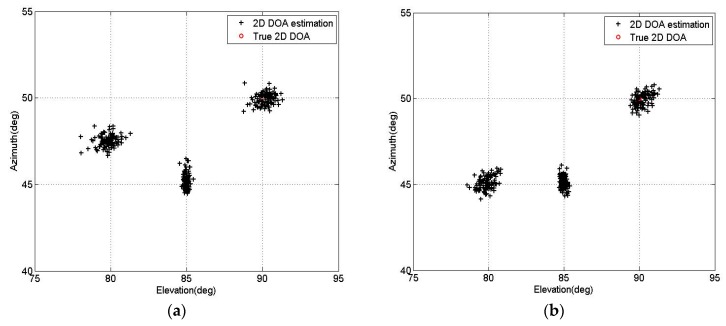
2D DOA Estimation results of proposed BCM-AMM algorithm. (**a**) Three signals with different elevation and azimuth angles; (**b**) Two signals with the same azimuth angles.

Figure 8a depicts the 2D DOA estimation results of proposed BCM-AMM algorithm with T = 200, SNR = 5 dB. We now keep the same elevation angles and change the azimuth angles to $\left[\beta_{1},\beta_{2},\beta_{3}]=[45{}^{\circ},45{}^{\circ},50{}^{\circ}\right]$. Figure 8b depicts the 2D DOA estimation results of the proposed BCM-AMM algorithm with T = 200, SNR = 5 dB. From two figures, the elevation and azimuth angles can be clearly observed and correctly matched even when two signals have the same azimuth angles.

**Figure 9:**
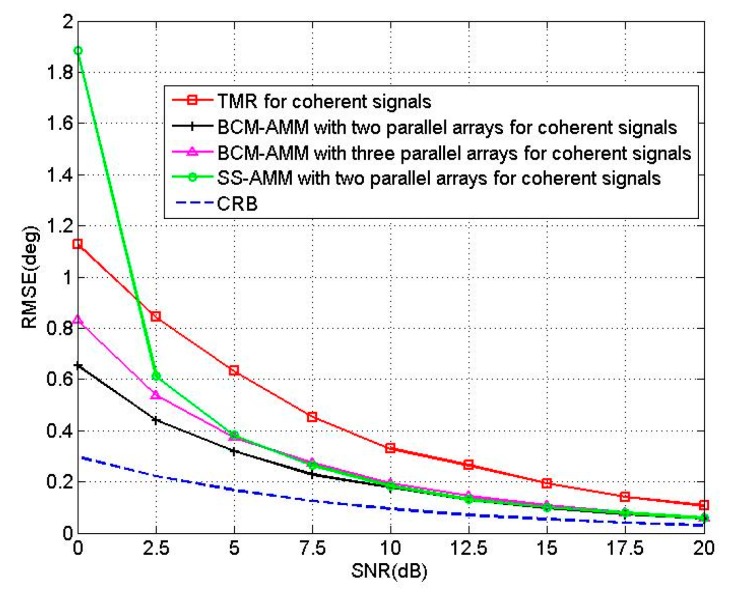
RMSE of four algorithms *versus* SNR.

**Figure 10:**
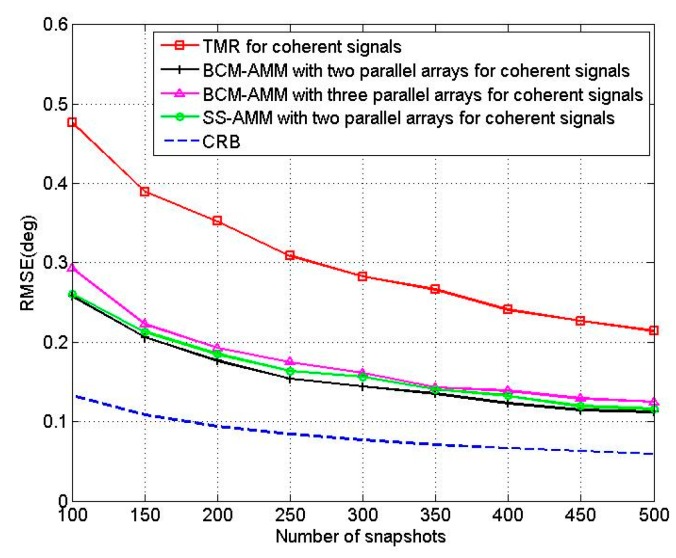
RMSE of four algorithms *versus* snapshots.

In the fifth experiment, we compare the BCM-AMM algorithm with TMR algorithm [25], SS-AMM and CRB. We called the algorithm that SS technology combines with AMM as SS-AMM. For the BCM-AMM algorithm, we use a two-parallel array with *M* = 15, *N* = 15 and a three-parallel array with *M* = 14, *N* = 8, respectively. For the TMR algorithm [25], we use two 15-element linear arrays. Two coherent sources are located at the angles of $\left[\theta_{1},\theta_{2}]=[60{}^{\circ},70{}^{\circ}\right]$, $\left[\beta_{1},\beta_{2}]=[50{}^{\circ},60{}^{\circ}\right]$. Figure 9 shows the RMSEs of the proposed BCM-AMM algorithm and TMR algorithm *versus* SNR with T = 200. Figure 10 shows the RMSEs of the proposed BCM-AMM algorithm and TMR algorithm *versus* snapshots with SNR = 10 dB. The two figures show that the estimation precision of the proposed BCM-AMM algorithms is higher than that of the TMR. Figure 9 and Figure 10 also show that the precision of BCM-AMM with two-parallel array is better than the BCM-AMM with three-parallel array for coherent signals. In addition, from Figure 9, we can find that BCM shows better performance than SS in the case of low SNR.

**Figure 11:**
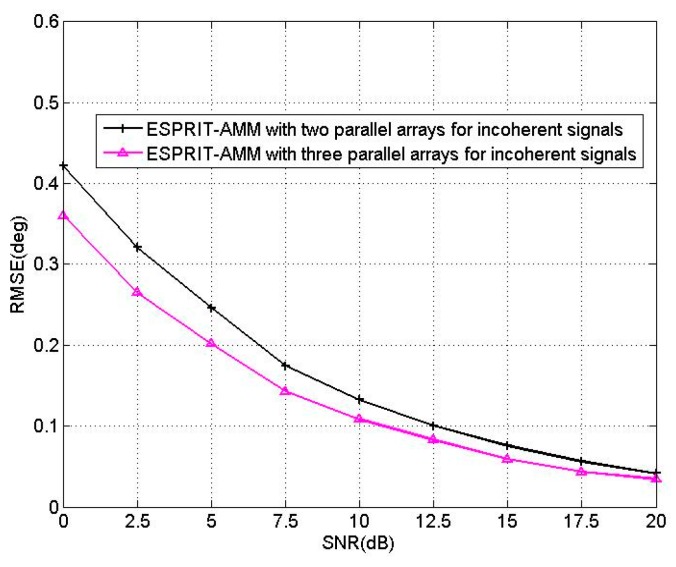
RMSE of the BCM/ESPRIT-AMM algorithms *versus* SNR.

In the sixth experiment, we consider two incoherent sources located at the angles of $\left[\theta_{1},\theta_{2}]=[60{}^{\circ},70{}^{\circ}\right]$, $\left[\beta_{1},\beta_{2}]=[50{}^{\circ},60{}^{\circ}\right]$. The RMSEs of BCM-AMM for two-parallel array and three-parallel array are provided. Since the two signals are incoherent, the ESPRIT-like algorithm is utilized to estimate elevation angles. Therefore, the BCM-AMM algorithm should be changed to ESPRIT-AMM algorithm. Figure 11 shows the RMSEs of proposed ESPRIT-AMM algorithms *versus* SNR with T = 200. From Figure 11, it is observed that the precision of ESPRIT-AMM with three-parallel array is better than that of the ESPRIT-AMM with two-parallel array for incoherent signals.

**Figure 12:**
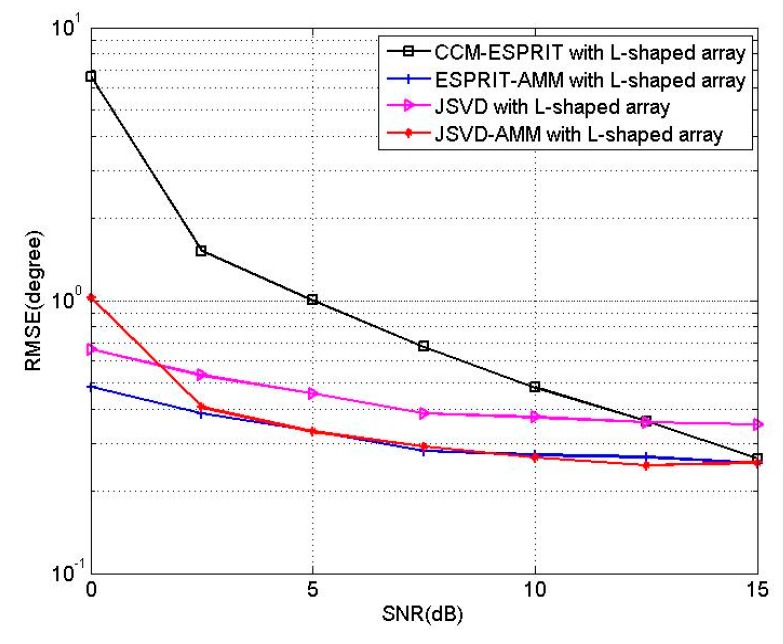
RMSE of four algorithms *versus* SNR.

**Figure 13:**
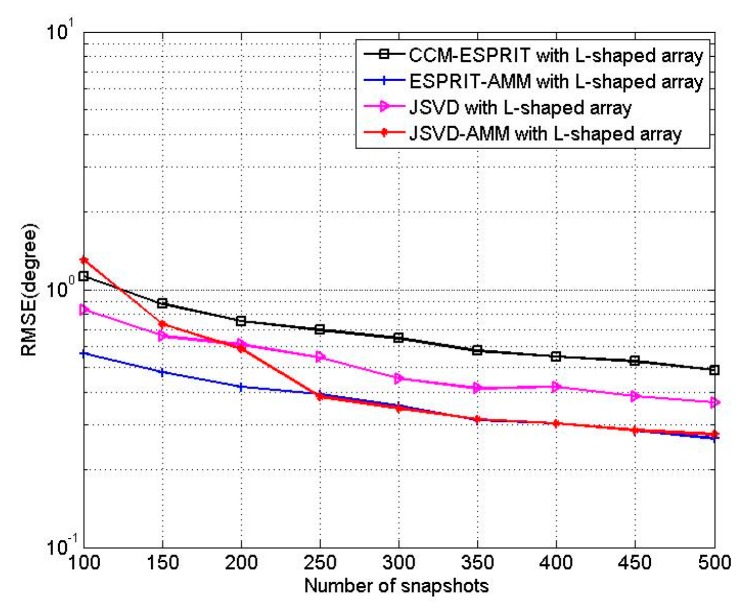
RMSE of four algorithms *versus* snapshots.

In the last experiment, we test the performance of JSVD-AMM and ESPRIT-AMM for L-shaped array. We consider an L-shaped array consisting of two 10-element linear arrays, and three incoherent sources located at the angles of $\left[\theta_{1},\theta_{2},\theta_{3}]=[40{}^{\circ},50{}^{\circ},60{}^{\circ}\right]$, $\left[\beta_{1},\beta_{2},\beta_{3}]=[20{}^{\circ},30{}^{\circ},40{}^{\circ}\right]$. Figure 12 shows the RMSEs of JSVD-AMM, ESPRIT-AMM, JSVD [9] and CCM-ESPRIT [17] *versus* SNR with T = 500. Figure 13 shows the RMSEs of JSVD-AMM, ESPRIT-AMM, JSVD [9] and CCM-ESPRIT [17] *versus* snapshots with SNR = 10 dB. From Figure 12, it is observed that the performance of JSVD-AMM is better than that of the JSVD with SNR > 2.5 dB and the performance of ESPRIT-AMM is better than CCM-ESPRIT with SNR < 15 dB. From Figure 13, it is observed that the performance of JSVD-AMM is better than that of the JSVD with snapshots >200 and the performance of ESPRIT-AMM is better than CCM-ESPRIT for different snapshots. But we should not neglect that JSVD-AMM and ESPRIT-AMM have obvious advantages in reducing complexity, which shows in Section 5.

## 7. Conclusions

In this paper, AMM methods are proposed for 2D DOA estimation for parallel linear arrays. Under the assumption that elevation angles are known *a priori* or estimated, the azimuth angles are estimated without EVD or peak search. Moreover, the azimuth angles are matched with the estimated elevation angles automatically. Compared with existing 2D DOA estimation algorithms, the advantages of the AMM methods are threefold. First, they can be used in conjunction with any existing 1D DOA estimation algorithms and the complexity is close to the used 1D DOA estimation algorithm. Second, they can achieve automatically paired 2D angles. Third, in the process of estimating azimuth angles, aperture loss is avoided for the coherent signal for the bilateral AMM algorithm.

## Notation

[•]^+^: Moore-Penrose generalized inverse
[•]^*T*^: transpose
[•]^*^: conjugate
[•]^*H*^: conjugate transpose
*E*[•]: statistical expectation
[**M**]_*i*,_: the *i*th row of matrix **M**
[**M**]_:,*i*_: the *i*th column of matrix **M**
[**v**]_*i*_: the *i*th element of vector **v**
**I**_*K*_: *K* × *K* identity matrix
